# Combinatorial Optimization of Cystine-Knot Peptides towards High-Affinity Inhibitors of Human Matriptase-1

**DOI:** 10.1371/journal.pone.0076956

**Published:** 2013-10-11

**Authors:** Bernhard Glotzbach, Michael Reinwarth, Niklas Weber, Sebastian Fabritz, Michael Tomaszowski, Heiko Fittler, Andreas Christmann, Olga Avrutina, Harald Kolmar

**Affiliations:** 1 Clemens-Schöpf-Institut für Organische Chemie und Biochemie, Technische Universität Darmstadt, Darmstadt, Germany; 2 AB SCIEX Germany GmbH, Darmstadt, Germany; Universidad de Granada, Spain

## Abstract

Cystine-knot miniproteins define a class of bioactive molecules with several thousand natural members. Their eponymous motif comprises a rigid structured core formed by six disulfide-connected cysteine residues, which accounts for its exceptional stability towards thermic or proteolytic degradation. Since they display a remarkable sequence tolerance within their disulfide-connected loops, these molecules are considered promising frameworks for peptide-based pharmaceuticals. Natural open-chain cystine-knot trypsin inhibitors of the MCoTI (*Momordica cochinchinensis* trypsin inhibitor) and SOTI (*Spinacia oleracea* trypsin inhibitor) families served as starting points for the generation of inhibitors of matriptase-1, a type II transmembrane serine protease with possible clinical relevance in cancer and arthritic therapy. Yeast surface-displayed libraries of miniproteins were used to select unique and potent matriptase-1 inhibitors. To this end, a knowledge-based library design was applied that makes use of detailed information on binding and folding behavior of cystine-knot peptides. Five inhibitor variants, four of the MCoTI family and one of the SOTI family, were identified, chemically synthesized and oxidatively folded towards the bioactive conformation. Enzyme assays revealed inhibition constants in the low nanomolar range for all candidates. One subnanomolar binder (K_i_ = 0.83 nM) with an inverted selectivity towards trypsin and matriptase-1 was identified.

## Introduction

**Figure 1:**
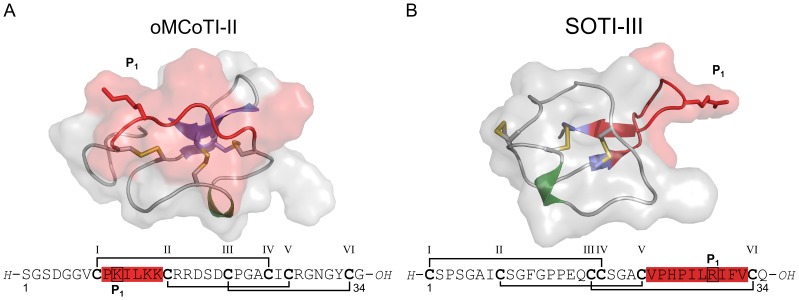
Sequences and structures of cystine-knot trypsin inhibitors. (**A**) Knottin oMCoTI-II (pdb: 1ha9). (**B**) SOTI-III (pdb: 4aor). Secondary structure is shown as cartoon with surface, and cysteine residues are depicted as yellow sticks; protease-binding regions (or inhibitor loops) are depicted in red, β-sheets - in blue, and α-helices - in green. Cystine-forming residues are marked bold, and the numbering of respective cysteines is according to their appearance in the sequence.

Cystine-knot peptides, often referred to as knottins, can be considered as one of Nature’s combinatorial libraries [1]–[4]. These peptides have been identified in various organisms, among them fungi, plantae, porifera, mollusca, arthropoda, and vertebrata. While they share a common fold, they display a notably large diversity within the primary structure of flanking loops that is also correlated with a diversity of biological activities [2]–[5]. Their amide backbone of about 30 to 40 amino acid residues is compacted by three disulfide bonds which form the characteristic mechanically interlocked structure [6]. Three β-strands linked through three disulfide bonds define their structural core, where the ring-forming connection of CysI to CysIV and CysII to CysV is penetrated by a third cystine between CysIII and CysVI (Figure 1) [1]–[4]. NMR measurements of dynamics of backbone NH groups revealed high structural rigidity [7]. Considering the extensive network of hydrogen bonds which permeates the inner core, especially *via* the *β-*strands, thus adding a substantial thermodynamic stability, the cystine-knot motif displays an exceptional structural and thermal robustness [8]–[10].

Trypsin inhibitors isolated from the bitter gourd *Momordica cochinchinensis* (MCoTI, Figure 1A) and the squirting cucumber *Ecballium elaterium* (EETI) are prominent members of the ICK (inhibitor cystine-knot) family. Both share the typical architecture of an ICK peptide with the functional loop comprising six amino acids located between CysI and CysII (Figure 1) [3], [11]. In contrast, recently reported miniproteins isolated from spinach *Spinacia oleracea* (SOTI I–III, Figure 1B) have shown no similarity to known plant protease inhibitors, but to antimicrobial peptides from the seeds of *Mirabilis jalapa* with the inhibitory loop located between CysV and CysVI (Figure 1) [12], [13]. Structural information is available for the members of both inhibitor families [13]–[17].

Sequence and structure alignments of members of a respective miniprotein family reveal a conserved structural core, while the surface-exposed loops possess a high flexibility in terms of primary structure [3]. Thus, through substitution of surface-exposed residues bioactive variants can be generated that can serve as tailor-made compounds for potential diagnostic and therapeutic applications [10], [18]–[20]. Several knottins have already been optimized by rational design or combinatorial library screening towards binding to targets of medical relevance [18], [21]–[32]. For example, a MCoTI-II-derived miniprotein comprising a non-native hydrazone macrocyclization motif was reported to simultaneously inhibit all four monomers of human mast cell tryptase β, a protease of clinical relevance related to allergic asthma [27], [28]. Several rounds of directed evolution and rational design of the scorpion-derived miniprotein Leiurotoxin I from *Leiurus quinquestriatus hebraeus* resulted in its enhanced binding to gp120 of the viral particle of HIV, thus inhibiting cell entry [25], [26], [33]. Furthermore, cancer-related integrins have been successfully labeled *in vivo* with radioactive ^64^Cu and ^111^In *via* selective targeting with knottins containing an integrin-binding RGD motif and used for PET (positron emission tomography) and SPECT (single-photon emission computed tomography) imaging [21]–[24].

Knottins are readily accessible both by recombinant production and SPPS (solid-phase peptide synthesis) [3]. Indeed, obvious difficulties arising upon on-support chain assembly can be easily overcome using the wide-ranging repertoire of modern peptide synthesis, and the crucial step, regioselective formation of a tridisulfide pattern, can be efficiently controlled using optimized oxidation conditions [3], [34].

Matriptase-1, a TTSP (type II transmembrane serine protease) of about 855 amino acids, belongs to the family of S1 trypsin-like proteases [35], [36]. It combines an amino terminal hydrophobic transmembrane region with an extracellular section of several domains, among them a trypsin-like catalytic and a low-density lipoprotein region [35]–[37]. Autocatalytic activation of the zymogen is assisted by its cognate inhibitor HAI-1 (hepatocyte growth factor activator inhibitor-1) and does not depend on other proteases. To date, the mechanism of autocatalytic activation has not been fully understood [35], [37]–[39]. Interestingly, matriptase-1 is also activated *via* acidification of the enzyme, therefore indicating its role in cellular acidosis [40]. Studies on knock-out mice have shown that matriptase-1 is essential for epidermal barrier functions, hence postnatal survival, as well as growth of hair follicles, and thymic homeostasis [41]. Moreover, matriptase-1 has been reported to be expressed not only in epithelial cells, but also in mast cells, B-cells, and blood monocytes [42]–[44]. Among its numerous substrates of which most are important for cell adhesion and tissue remodeling, processing of pro-uPA (pro-urokinase plasminogen activator) and pro-HGF (pro-hepatocyte growth factor) have been shown to be significantly involved in tumor growth and metastasis [45]. Expression rates of matriptase-1 were reported to reflect the degree of tumor progression in several types of cancerous cells, thus indicating a crucial role of this protease in tumor metastasis [46]–[48]. This was evidenced through various experiments, both *in vitro* and *in vivo*, in which the enzyme was inhibited [35], [49]–[51]. The ratio of matriptase-1 and HAI-1, which is shifted towards matriptase-1 in cancer cells, is of major importance for tumor invasiveness [45], [52], [53]. Moreover, matriptase-1 has been reported to be implicated in a number of other diseases, among them osteoarthritis and atherosclerosis, and to induce cancer itself [42], [54], [55]. In conclusion, matriptase-1 has become a promising target for drug development. To date, only one peptide-based inhibitor of matriptase-1 with a picomolar K_i_ has been reported [56], [57]. Despite its excellent inhibition constants against matriptase-1, this four-amino-acid peptide with the sequence *H*-R-Q-A-R-*Bt* (Bt stands for carboxy terminal benzothiazole substituent) displays a low selectivity. Since for *in vivo* experiments a high selectivity and serum half-life are indispensable, this inhibitor presumably is not suitable for experiments towards tumor targeting *in vivo*. Here we describe the isolation of selective cystine-knot peptides of high affinity from knowledge-based combinatorial miniprotein libraries and their functional characterization *in vitro* and in cell culture.

## Materials and Methods

### Media and Reagents

All media were prepared as previously reported [18], [58], [59]. YPD medium contained 20 g/L peptone, 20 g/L dextrose, and 10 g/L yeast extract. Selective SD-CAA medium incorporated 6.7 g/L yeast nitrogen base without amino acids, 20 g/L dextrose, 8.6 g/L NaH_2_PO_4_·H_2_O, 5.4 g/L Na_2_HPO_4_, and 5 g/L Bacto casamino acids. SG-CAA medium was prepared similarly except for the addition of 100 mL/L polyethylene glycol 8000 (PEG 8000) and the substitution of dextrose by galactose. DYT medium contained 10 g/L yeast extract, 16 g/L tryptone, 5 g/L and 100 mg/L ampicillin. Phosphate-buffered saline (PBS) was composed of 8.1 g/L NaCl, 0.75 g/L KCl, 1.13 g/L Na_2_HPO_4_, and 0.27 g/L KH_2_PO_4_ at pH 7.4.

RPMI cell culture media (with and without phenol red) was supplemented with 10% (v/v) fetal calf serum (FCS) and antibiotics. These materials were purchased from Sigma-Aldrich.

Human matriptase-1 was produced recombinantly, autocatalytically activated and purified as previously reported [35], [49], [51], [60]. Bovine pancreatic trypsin, thrombin and uPA were purchased from Sigma-Aldrich and Hepsin from R&D Systems.

### Variant Cloning and Library Synthesis

For the initial display experiments of SOTI-III *wild type* and the yeast libraries based on the MCoTI-II and SOTI-III scaffold the encoding gene fragments were amplified by PCR with Taq polymerase with the use of primers with 50-bp overlap to the pCT plasmid up- or downstream of the *Nhe*I and *Bam*HI restriction sites, respectively. Positions for randomization in case of the SOTI-III library contained the NNK degenerate codon. For the MCoTI-II library, weighted randomization of respective residues was achieved upon synthesis using pre-made codon mixtures as described [61]. Amplified PCR products were purified by phenol/chloroform extraction. The vector was digested with *Nhe*I and *Bam*HI and purified *via* sucrose density gradient for homologous recombination in yeast. For the electroporation reaction 1–4 µg of linearized plasmid and 10–12 µg of insert were used [58]. After 1 h incubation (YPD medium, 30°C) library size was estimated by dilution plating. The yeast cells were transferred into selective SD-CAA medium, grown at 30°C to OD_600_ = 10–12 and split into new SD-CAA medium. Library stocks were stored at –80°C [58]. Yeast cells were induced in SG-CAA medium (starting OD_600_ of 0.1–0.2, 20°C, 48 h, 220 rpm).

### Surface Binding Assays and Library Screening

Surface presentation of miniproteins was monitored by flow cytometry. 1·10^7^ cells were labeled consecutively with 1∶20 dilutions of anti-cMyc antibody (monoclonal, mouse, Abcam), anti-mouse IgG biotin conjugate (polyclonal, goat, Sigma-Aldrich), and Streptavidin, R-phycoerythrin conjugate (SPE) for 10 min on ice.

Protease binding assays and one-dimensional screenings of recombinant knottin libraries were conducted by incubation of knottin-presenting yeast cells with the respective biotinylated protease for 30 minutes on ice. Subsequently, the cells were resuspended in a 1∶20 dilution of SPE for 10 min. The cells were analyzed in an Accuri C6 (Becton Dickinson) or were sorted using a MoFlo cell sorter. Sorting parameters were: trigger side scatter 650, PMT FL2 600, ex. 488 nm filter FL2 570/40. FCS files were analyzed using CFlow software or Summit 4.3, respectively.

For two-dimensional screening the yeast cells were consecutively incubated for 30 min at 0°C with 1∶20 dilutions of each anti-cMyc antibody containing the desired concentration of biotinylated protease as well as a mixture of SPE and anti-mouse-IgG FITC (parameters: trigger side scatter 650, FL1 600, FL2 600).

Approximately 2×10^8^ yeast cells were run through the flow cytometer at the first round of sorting. The selected cells were cultured after each screening round in SD-CAA medium. Next screening rounds were performed with at least 10 times the number of yeast cells collected in the previous round to ensure library diversity. Sort stringency was increased by reducing the protease concentration in subsequent screening rounds.

Plasmid DNA from positive clones was isolated and transformed into DH5α competent *E. coli* cells for plasmid amplification. DNA sequencing was performed using the oligonucleotide pCT-seq-lo.

### Cell Inhibition Assay

Human prostate cancer cells (PC-3, Merck KGaA) were cultured in DMEM medium with 10% FCS at 37°C and 5% CO_2_, washed with cation-free PBS and harvested by scraping. 1×10^5^ cells were incubated in presence of 250 µM Bz-*β*-Ala-Gly-Arg-*p*NA·AcOH (American Diagnostica), which is a specific inhibitor of urokinase, and the inhibitor of interest in defined dilutions overnight. Product formation was monitored at 405 nm before and after incubation in a microplate reader. IC_50_ was calculated by non-linear regression using SigmaPlot 11.

### Synthesis of Cystine-knot Miniproteins

Peptides were assembled using standard Fmoc-SPPS chemistry on a fully automated microwave-assisted CEM *Liberty*® peptide synthesizer. Peptide acids were generated using an Fmoc-Gln-preloaded TentaGel resin, whereas peptide amides were synthesized on a *ChemMatrix* Fmoc-Rink amide resin. After cleavage from the solid support, oxidative folding was conducted as recently reported [34]. About 40 mg of the corresponding lyophilized crude peptide were suspended in 500 µL acetonitrile and treated in an ultra-sonic bath for 5 min. Afterwards, 3500 µL of the folding mixture consisting of 10% (*v*/*v*) DMSO, 10% (*v*/*v*) TFE and guanidinium hydrochloride (GuHCl) (1 M) in aqueous sodium phosphate buffer (50 mM, pH 7) were added [34]. Reaction progress was monitored *via* analytical HPLC and ESI-MS (Figures S7 and S8) [34]. For termination of the reaction and purification of the bioactive miniprotein, the mixture was directly injected into a semi-preparative HPLC system.

### RP-HPLC, LC-ESI-MS, and CD Spectroscopy

Analytical RP-HPLC was performed using a Varian LC 920 system equipped with a *Phenomenex* Synergi 4 µ Hydro-RP 80 Å (250×4.6 mm, 4 µm) column applying linear gradients of acetonitrile at a flow rate of 1 mL/min. Semi-preparative RP-HPLC purifications were performed using a Varian LC 940 system equipped with an axia-packed *Phenomenex* Luna C18 (250×21.2 mm, 5 µm, 100 Å) column applying linear acetonitrile gradients at a flow rate of 18 mL/min. Isocratic elution (10% eluent B over 2 (on analytical scale) or 5 min (on semi-preparative scale)) was followed by a linear gradient of 10→60% B (for MCoTI variants) or 10→80% B (for SOTI variants) over 20 min, respectively.

LC-MS was performed with a *Shimadzu* LC-MS 2020 equipped with a *Phenomenex* Jupiter C4 (50×1 mm, 5 µm, 300 Å) column using linear acetonitrile gradients at a flow rate of 0.2 mL/min (Figures S7 and S8). Isocratic elution (2% eluent B over 2 min) was followed by a linear gradient of 2→100% B over 10 min. Cystine-knot disulfide bond topology of MCoTI Var. 4 was confirmed using MS^3^-technology (AB Sciex, 4000 QTRAP® LC/MS/MS System; data not shown).

CD spectroscopy was performed as previously reported [34]. The peptides were dissolved in 2 mM aqueous Na_2_HPO_4_ (pH 7) to a final concentration of 50 µM. The resulting spectra (Figure S9 and S10) were obtained through accumulation of 10 spectra each, using a 0.1 mm quartz cuvette at 0.5 nm steps.

### Inhibition Assays

Protease inhibition assays which resulted in substrate-independent inhibition constants were performed as previously described [11], [13], [34], [62].

Measurements were carried out in triplicate using a *Tecan Genios* ELISA reader. The normalized residual proteolytic activity (v/v_0_) of proteases was determined using substrates Boc-QAR-*p*NA (250 µM, (pNA stands for *para*-nitro aniline), Boc-QAR-AMC (250 µM, AMC stands for amino-methyl coumarin) or Spectrozym tPA (250 µM, American Diagnostica, CH_3_SO_2_-D-CHT-Gly-Arg-pNA AcOH). Product formation was monitored after preincubation (30 min, RT) with inhibitor at different concentrations over 30 min by measuring the absorbance at 405 nm for *p*NA substrates or the fluorescence emission for AMC substrates (ex. 360 nm, em. 465 nm), respectively. Selectivity data were carried out in duplicates with final protease concentrations of uPA and thrombin of 5 nM. In case of hepsin 50 mM Tris/HCl pH 9.0 was used as assay buffer.

Apparent inhibition constants (K_i_^app^) were calculated by fitting the Morrison equation (1) for tight-binding inhibitors to the relative reaction velocity using non-linear regression (Marquardt-Levenberg algorithm, Sigma Plot 11) [63].
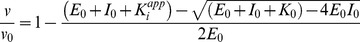
(1)

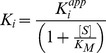
(2)


Substrate-independent inhibition constants K_i_ were calculated from K_i_^app^ and K_m_ of the enzyme according to (2). The Michaelis-Menten constant K_m_ for the substrates and proteases were determined previously [49], [64].

## Results

### Selection of Knottin Scaffolds

Since the overall structure of matriptase-1 is similar to trypsin, the preference for cleavage at basic residues at the P_1_ position is maintained [49]. Hence, we considered trypsin-inhibiting miniproteins as a starting point for functional combinatory library design to isolate inhibitors of matriptase-1 [17]. From the plethora of miniproteins that are characterized to date, scaffolds were selected matching the following criteria: inhibitor of a trypsin-like protease, known three-dimensional structure, tolerance to variation of loop lengths and sequence, known mechanism of folding and disulfide bond formation, as well as availability through chemical and recombinant routes of synthesis [2]–[4]. Two different scaffold proteins have been selected based on the aforementioned requirements. The first selected scaffold was based on the spinach-derived inhibitor SOTI-III. The structure of this protease inhibitor has been recently elucidated by X-ray crystallography [13]. Since the inhibitor loop of SOTI-III is located between CysV and CysVI, this miniprotein is structurally and sequentially very distinct to MCoTI-II, which was chosen as second scaffold (Figure S1). This scaffold is based on miniproteins from the seeds of the squash plant *M*. *cochinchinensis*. This plant produces a number of miniprotein-based trypsin inhibitors, both backbone-cyclized macrolactams and variants lacking this motif (so-called ‘open-chain’ variants), which are slightly different in their sequences [1], [17]. To evaluate which of the natural MCoTI variants could serve as a scaffold for the generation of matriptase-1 inhibitors, natural inhibitors were isolated form the *M*. *cochinchinensis* seeds using known extraction procedures followed by HPLC separation (Figure S2) [17], [65]. Miniproteins from various fractions were identified by ESI-MS and examined for inhibition of matriptase-1 (Table S1). MCoTI-II, a macrolactam-cyclized miniprotein consisting only of natural amino acids, was found to be the most efficient natural inhibitor of matriptase-1 and therefore chosen as starting scaffold. Synthetic open-chain MCoTI-II (oMCoTI) displayed a K_i_^app^ similar to that of its cyclic counterpart (Table S1). Interestingly, SOTI-III is a less potent inhibitor of trypsin and did not display measurable inhibitory activity against matriptase-1 (Table 1).

**Table 1:** Inhibition constants of inhibitors studied in this work.

Inhibitor	K_i_ (Trypsin) (nM)	K_i_ (Matriptase-1) (nM)
SOTI-III *wt*	60.6±8.4	>1000
SOTI Var. 1	>1000	28.9±3.5
MCoTI-II *wt*	2.37±0.96	80.7±10.0
MCoTI Var. 1	31.7±4.3	4.4±0.6
MCoTI Var. 2	19.2±2.8	3.3±0.4
MCoTI Var. 3	22.3±3.0	7.8±1.0
MCoTI Var. 4	35.8±4.7	0.83±0.14
S1^a^ [51]	118±16	7.1±0.87
S2^a^ [51]	544±74	28.2±3.5

a Structural information for reference compounds S1 and S2 are depicted in Figure S3.

### SOTI-III-based Library Screening

**Figure 2:**
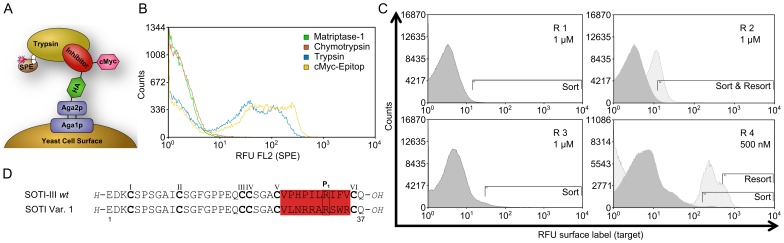
Yeast surface display of SOTI-III *wild type* and screening against matriptase-1. (**A**) Schematic illustration of Aga1p/Aga2p surface-displayed inhibitor (red) flanked by the amino terminal HA (Human influenza hemagglutinin) epitope (green) and the carboxy terminal cMyc epitope (purple). Functional display of the inhibitor is monitored by incubation with biotinylated trypsin followed by fluorescence labeling with streptavidin, R-phycoreythrin conjugate (SPE). (**B**) FACS histogram overlay of yeast surface presented SOTI-III *wild type* labeled with anti-cMyc antibody (yellow), trypsin (blue), matriptase-1 (green) and chymotrypsin (brown). (**C**) FACS overlays of matriptase-1 binder enrichment. The sorting round (R) and the matriptase-1 concentration used in each round (µM) is given in the figures. Dark grey: FACS histogram during sorting. Light grey: FACS histogram during resort (only rounds 2 and 4). (**D**) Sequence alignment of SOTI-III *wild type* and matriptase-1-binding SOTI variant 1. Randomized residues are colored in red. Cysteines are depicted in bold letters, while cystine connections are omitted for clarity.

To obtain knottin-based matriptase-1 binders, yeast surface display was chosen as its applicability to the screening of cystine-knot-based peptide libraries has been already demonstrated [22], [58], [59], [66]. To this end, the SOTI-III *wild type* or library-encoding DNA was genetically fused to the *Saccharomyces cerevisiae* Aga2p coding sequence. The resulting constructs are under control of the galactose promoter [22]. Induction with galactose yields a fusion protein consisting of Aga2p, a glycine-serine linker, an HA-epitope, the miniprotein, and a cMyc epitope (Figure 2A) [58], [66]–[68]. The fusion is covalently bound to the surface-anchored Aga1p [58], [66]. Functional display of SOTI-III *wt* was shown by binding of biotinylated bovine pancreatic trypsin followed by flow cytometric analysis (Figure 2B). After verification of functional display of the *wild type* miniprotein, the inhibitor loop was randomized by PCR using oligonucleotides with NNK codon randomization (materials and methods section). All ten loop residues of SOTI-III were considered for full randomization including the P_1_ residue arginine, since for optimized matriptase-1 binding the P_1_ residue may be shifted to another position within the inhibitor loop. The resulting miniprotein library had a clonal diversity of 2×10^8^.

To isolate matriptase-1-binding SOTI-III variants, four consecutive fluorescence-activated cell sorting (FACS) screening rounds were performed (Figure 2, Figure 2C for flow cytometry screening, and materials and methods section) and one dominant clone was isolated after four sorting rounds (Figure 2D).

Subsequently, an alanine scan was conducted to determine the essential arginine residues within the inhibitor loop that contained four arginine residues. As a result, Arg29 and Arg32 were found imperative for binding and bioactivity, while Arg30 and Arg35 were dispensable without major loss of binding (Figure S4). The SOTI-based cystine-knot peptide Var. 1 was synthesized chemically using microwave-assisted Fmoc-SPPS followed by oxidative folding and HPLC purification yielding the bioactive peptide (Table S2). Subsequent inhibition assays revealed the inhibition constants against matriptase-1 and trypsin as 28.9 nM and >1 µM, respectively (Table 1).

### MCoTI-II-based Library Design

**Figure 3:**
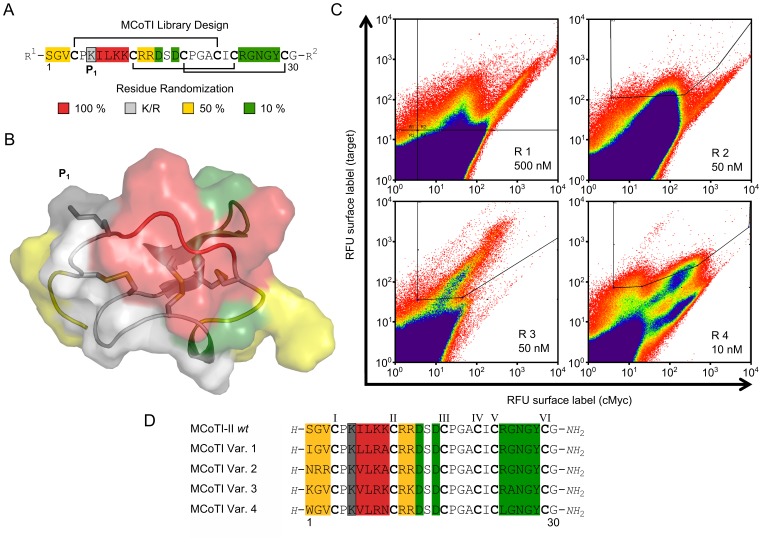
Summary of MCoTI-II-based library design and screening against matriptase-1. (**A**) Sequence of open-chain MCoTI-II *wild type*. Cysteines are depicted in bold letters. R^1^ represents the amino-terminal flanking sequence, including the HA-epitope. R^2^ represents the carboxy-terminal flanking sequence, including the cMyc-epitope. Codon randomization for (**A**), (**B**), and (**D**) as indicated by color (at pos. 6 only Lys or Arg was allowed, grey). (**B**) Secondary structure of MCoTI-II is shown as cartoon with surface, cysteine residues are shown as orange sticks. (**C**) FACS histograms showing four rounds of sorting with decreasing target concentration for enrichment of matriptase-1 binders. R denotes the sort round with the concentration of matriptase-1 indicated. Actual sort gates are shown. (**D**) Sequence alignment of matriptase-1-binding MCoTI variants. Cysteines are numbered according to the appearance in the sequence and depicted in bold letters, while cystine connections are omitted for clarity.

Encouraged by these promising results, we further optimized the library design towards more potent cystine-knot inhibitors of matriptase-1. A codon-based randomization of the oMCoTI-II scaffold was used for library generation (Figure 3A and 3B), which included the inhibitor loop and neighboring residues that may contribute to target binding [61]. It is well known that a proline is required at position P_2_ (amino-terminal to P_1_, Figure 1) of the inhibitor loop [2], [4]. Thus, Pro5 was not modified since it is essential for the formation of the six-residue canonical inhibitor loop conformation that is found in many protease inhibitors [1], [2], [4]. Codon 6 was randomized to code for Arg or Lys (50% each), and positions 7–10 were randomized to code for the full set of 19 canonical amino acids, excluding cysteine, using a codon-based randomization scheme (Figure 3A) [22]. In addition, neighboring residues were also included into the variegation scheme to enable improved subsite binding that may contribute to both enhanced affinity and specificity. Since these residues outside the inhibitor loop may be of relevance for oMCoTI-II folding and stability, simultaneous full randomization was avoided by maintaining the original residue at each position for 50% of the variants. As a consequence, in approximately 3% of the variants all five original amino acids that are located adjacent to the inhibitor loop are expected to be preserved and the average number of residue replacements was expected to be 7 (Figure S5). In oMCoTI-II, the carboxy-terminal loop is located adjacent to the inhibitor loop and therefore can affect target binding. Tolerance of this loop region towards amino acid exchanges has been extensively investigated for the structurally similar knottin EETI [18], [29], [69], [70]. This loop region is thought to be involved in the early folding process of the miniprotein via formation of a type II β-turn [2], [4], [11], [18], [70]. Since this loop sequence is a folding determinant, only moderate sequence variations were included by randomizing each position to 10%. Thus, over 50% of the variants can be expected to have none or one amino acid exchange within that region (Figure S5). The same moderate mutagenesis scheme was applied for D14 and D16 that are conserved in the ICK family of miniproteins and are involved in stabilization of the oMCoTI scaffold [2], [4]. As the active site of matriptase-1 is negatively charged, it may be beneficial for binding to allow replacement of these residues [49]. Overall, the randomization scheme applied here includes 17 out of 30 residues. However, on average only 6 to 8 of the 17 residues are expected to be changed in each variant and four of these are most likely located within the inhibitor loop.

### MCoTI-II Library Screening

To evaluate the feasibility of library design that includes 17 of 30 residues in the randomization scheme, two relatively small yeast libraries with a diversity of 2×10^6^ and 2×10^7^ clones, respectively, were independently constructed from the same synthetic library DNA and screened separately. After two to four rounds of screening, matriptase-1-binding populations were enriched. Individual matriptase-1-binding clones were identified using flow cytometry (Figure 3C). DNA sequences were obtained (10 from the screen of the library with a diversity of 2×10^6^ clones as well as 12 of the 3^rd^ and 16 out of the 4^th^ round of the library containing 2×10^7^ clones, respectively; Figure S6). From these, four binders were selected for detailed investigations (Figure 3D) that were independently identified several times in screening rounds three and four or displayed high affinity binding upon yeast cell surface affinity titration (Figure S6).

To determine the inhibition constants, chemical synthesis and oxidative folding of the putatively inhibiting cystine-knot peptides were performed as previously reported (Table S2) [34]. Correct fold of the miniproteins was proven through bioactivity, since it is known that knottins of the ICK family displaying an incorrect disulfide connectivity show a decreased inhibitory efficiency [3], [11], [15], [30], [34]. Moreover, CD spectra of SOTI *wt*, SOTI Var. 1, MCoTI *wt* and MCoTI Var. 4 indicated β-sheet formation (Figures S9 and S10). Disulfide bond connectivities were confirmed by MS^3^ mass spectrometry for MCoTI Var. 4 via continuous injection of a 3 µM solution of the miniprotein at a flow rate of 10 µL/min into an ABSCIEX 4000 QTRAP® LC/MS/MS system (data not shown) [71]. Inhibition constants in the low nanomolar to sub-nanomolar range were obtained for all MCoTI-based miniproteins (Table 1). An additionally performed selectivity study for the best MCoTI-based inhibitor candidate Var. 4 revealed inhibition constants K_i_ >10 µM against thrombin, uPA, and hepsin (Table 2). Moreover, inhibitory activity for matriptase-1 was approximately fortyfold higher than for trypsin (Table 1).

**Table 2:** Selectivity profile of MCoTI-based inhibitor Var. 4.

Protease	K_i_ (nM)
Trypsin	35.8±4.7
Matriptase-1	0.83±0.1
Thrombin	>10000^a^
Urokinase	>10000^a^
Hepsin	>10000^a^

a No inhibition was observed at 10 µM inhibitor concentration.

### Inhibition of uPA Activation

Urokinase-type plasminogen activator (uPA) causes the degradation of the extracellular matrix and plays a critical role in tumor invasion and metastasis [72], [73]. It was shown that activation of receptor-bound pro-uPA is affected by matriptase-1, which results in a decreased ability of uPAexpressing tumor cells to invade an extracellular matrix layer upon inhibition of membrane-bound matriptase-1 [72]. To investigate the inhibitory activity of the newly isolated matriptase-1 inhibitors on pro-uPA activation, a dose-response assay of uPA activity was performed in cell culture with SOTI-based variant (Var. 1) and the most potent MCoTI-based inhibitor (Var. 4) on human prostate carcinoma cancer cells (PC-3), as a upregulation of matriptase-1 expression level has been reported for this cell line [45], [69], [72].

**Figure 4:**
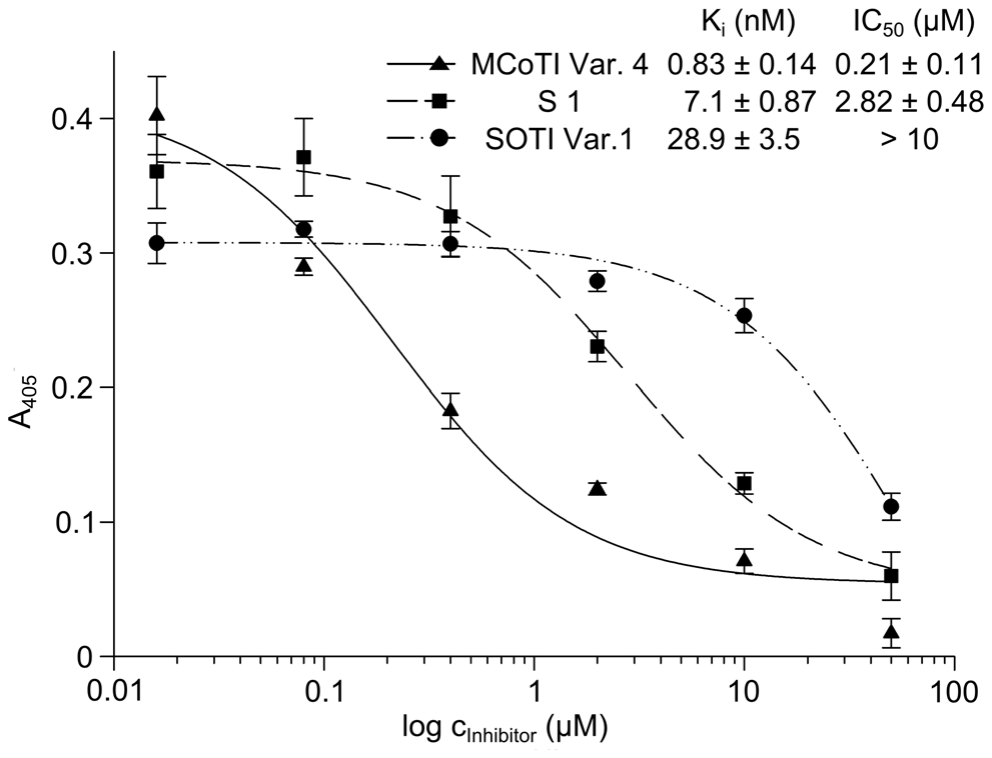
Inhibition assay of uPA activation by matriptase-1 on the surface of PC-3 cells. Depicted is the logarithmic inhibitor concentration against the absorption at 405

For the indirect determination of the IC_50_ of SOTI Var. 1 and MCoTI Var. 4 on the surface of these cancer cells, the turnover of an uPA substrate was monitored. Pro-uPA is activated through non-inhibited matriptase-1 and substrate turnover was measured and compared to the previously reported small molecule inhibitor S1 of matriptase-1 (Figure 4) [51]. In this experimental setting, the MCoTI-based inhibitor Var. 4 (K_i_ = 0.83 nM) exhibited an IC_50_ of 213 nM, while SOTI-III derived inhibitor Var. 1 displayed only minor activity. S1 a small-molecule inhibitor (Figure S3) that has been identified recently as potent matriptase-1 inhibitor with an K_i_ in the single digit nanomolar range was used as reference compound that displayed an tenfold higher IC_50_ value than MCoTI-based inhibitor Var. 4 in this assay [51]. For control SOTI *wt* was also applied in this experimental setting at a concentration of 10 µM, displaying no inhibition of either matriptase-1 or uPA.

## Discussion

For the isolation of miniprotein-based inhibitors by combinatorial library screening the design of the variant library is a crucial step. We chose a knowledge-based strategy that takes into account the expected contribution to target binding, as well as the natural variability and the contribution to structure and folding of each residue at each position. While we followed a classical variegation scheme for SOTI-III with a full randomization that is restricted to the carboxy terminal loop, a position-specific randomization scheme was applied for oMCoTI-II.

Screening of the SOTI-III library resulted in the isolation of a variant that displayed 29 nM K_i_ with respect to matriptase-1 inhibition and contained the sequence motif RRAR in the inhibitor loop. This agrees with the consensus sequence for matriptase-1 substrates and the highly potent inhibitor peptide *H*-R-Q-A-R-*Bt* [45], [49], [56], [57]. Despite the fact that the absolute position of the P_1_ arginine residue within the inhibitor loop remained unchanged, increased inhibitory activity towards matriptase-1 interestingly led to the total loss of trypsin inhibition. Hence, in comparison to the *wild type* miniprotein, the isolated SOTI-based matriptase-1 inhibitor (Var. 1) showed improved inhibitory activity and selectivity. Notably, the recently reported crystal structure of SOTI-III *wild type* revealed that out of the 10 carboxy terminal loop residues only 8 were in direct contact to trypsin [13]. Thus, exclusion of core-forming residues from the randomization scheme of SOTI-based inhibitor Var. 1 and generating a sub-library for the flanking residues might result in variants with further improved binding characteristics. Moreover, experiments on co-crystallization of matriptase-1 and SOTI Var. 1 are required to understand the mode of interaction of protease and inhibitor. Additionally, it would be beneficial to gain further knowledge on whether the conformational constraints that are imposed on the SOTI-III *wild type* inhibitor loop *via* integration into the cystine-knot scaffold are also conserved in the matriptase-1 inhibitor Var. 1. Assuming an unchanged mode of action, the knottin-based peptide acts as a matriptase-1 inhibitor rather than as a substrate that is readily and irreversibly cleaved.

Screening of the oMCoTI-scaffold-derived library resulted in several inhibitors that all displayed K_i_ values in low nanomolar to sub-nanomolar range. Despite the fact that the library diversity was 10-fold lower than for the SOTI scaffold and with 2×10^7^ clones relatively small, more potent binders were isolated corroborating the concept of knowledge-based library design. It should also be noted that oMCoTI-II *wt* in contrast to SOTI-III *wt* already displayed inhibitory activity against matriptase-1 and therefore may be the more suitable scaffold for optimization towards matriptase-1-binding and inhibition. Branched aliphatic residues were observed at the P_1’_ position of isolated matriptase-1-binding oMCoTI library variants, while leucine was the preferred P_2’_-positioned amino acid. In contrast to reported substrates and inhibitors, lysine was obviously favored over an arginine residue at the P_1_ position (Figure 3D) [45], [56]. While amino acid residue 1 displayed a large variability, replacements of the ‘GV-motif’ at positions 2 and 3 rarely occurred, demonstrating the importance of these residues for binding and/or folding (Figure 3D). Whereas no substitutions within loop 2 (flanked by CysII and CysIII) were observed, Arg24 was exchanged for leucine in the most potent inhibitor Var. 4 (Figure 3D). It remains to be elucidated, whether this residue replacement contributes to enhanced inhibition.

To investigate the inhibition of pro-uPA activation cell culture upon matriptase-1 inhibition, miniproteins SOTI Var. 1 and MCoTI Var. 4 as well as reference compound S1 were applied to human pancreatic PC-3 cells (Figure 4) [45], [72]. MCoTI-based knottin Var. 4 that had a subnanomolar K_i_ towards matriptase-1 also displayed the lowest IC_50_ with respect to the inhibition of proteolytic activity in a PC-3 cell line [74]–[76]. This indicates that inhibitor-mediated reduction of matriptase-1 activity contributes to the decrease of uPA activity. IC_50_ values ranged from a nanomolar to micromolar range and MCoTI-based inhibitor Var. 4 was found to be 10-fold more potent than recently described peptidomimetic small-molecule inhibitors (Figure 4) [51].

All three inhibitors investigated displayed IC_50_ values of protease inhibition on PC-3 cells more than 100-fold higher compared to their K_i_ of matriptase-1 inhibition. This discrepancy may arise from the complicated situation in cell culture since matriptase-1 activity is regulated by the cognate natural tight-binding inhibitor HAI-1. Co-expression of HAI-1 and matriptase-1 suppresses matriptase-1 proteolytic activity. Interestingly, HAI-1 has also been considered to be required for activation of matriptase-1 and to be involved in its expression and autoprocessing [38], [39], [41], [57], [77]. Moreover, absence of HAI-1 seems to cause rapid turnover of active matriptase-1 [57], [77]. Hence, the complicated conditions in the cell-culture media, in the cell and on its surface may account for the observed differences of K_i_ and IC_50_.

In recent years, matriptase-1 has attracted keen scientific interest as a target for the development of inhibitors. Steinmetzer and coworkers reported small molecule inhibitors that display similar potency and selectivity *in vitro* as well as in cell-based assays as the miniproteins generated in this study [51], [76]. In addition, two types of peptidic matriptase-1 inhibitors have been identified to date [49], [56]. The short substrate-derived inhibitor *H*-R-Q-A-R*-Bt* displays an inhibition constant in the double-digit picomolar range. Due to the small size and susceptibility to proteolytic degradation, *in vivo* half-life can be expected to be short. Moreover, the universal sequence is not selective for matriptase-1, but inhibits various proteases in the pico- to nanomolar range [56]. Compared to the tetrapeptide, the sunflower trypsin inhibitor (SFTI)-based matriptase-1 inhibitor that has been described recently has an increased size (14 residues) combined with a constrained structure, thus being potentially more stable and applicable for *in vivo* experiments [49]. Recently, Daly and coworkers obtained by rational design and positional scanning mutagenesis a cyclic MCoTI-II variant with subnanomolar inhibition constant of matriptase-1 that was found to be more potent than SFTI derived variants, corroborating the notion that the McoTI-II miniprotein scaffold provides an excellent structural environment for the development of potent and selective matriptase inhibitors [78]. Serum stability and potential oral availability has been shown for several knottins and it will be interesting to see whether the MCoTI-derived inhibitors display similar stability in cell culture and serum while maintaining activity and selectivity.

Knottins have been introduced by Cochran and coworkers as a new class of agents for imaging of tumor marker expression in living systems [18], [24], [79]. For example, ^64^Cu-DOTA-conjugated knottin peptides were stable in mouse serum, and *in vivo* metabolite analysis showed minimal degradation in blood or tumor rendering this type of stable peptides very promising candidates as clinical diagnostics for a variety of cancers [79]. The spectrum of tumor targeting knottins, which is currently restricted to cystine-knot peptides containing integrin binding RGD sequences in their binding loops, can be extended by matriptase-1 binders for imaging applications. The miniproteins described in this study selectively detect cell-surface-exposed and enzymatically active matriptase-1 on tumor cells that is not complexed with the natural inhibitor HAI-1. In contrast, with one notable exception most antibodies cannot distinguish between the active and inactive form of matriptase-1, due to their binding to accessible epitopes that are not linked to the active site or conformational changes upon activation. Recently, Craik and coworkers showed that an active-site-specific, recombinant human antibody for matriptase-1 can be used to visualize the tumorigenic epithelium using near-infrared and single-photon emission computed tomography imaging, corroborating the notion that the active form of matriptase-1 is a tumorigenic biomarker [81]. Since matriptase-1 provides the major contribution to tumor invasion and progression, knottins selectively addressing the active site of matriptase-1 may become valuable tools for tumor imaging, particularly for the prediction of tumor invasiveness.

## Conclusions

To conclude, we have proven the applicability of a knowledge-based miniprotein library design to the development of potent inhibitors of human matriptase-1 using a codon-based, weighted, and selective randomization scheme. A set of cystine-knot miniprotein variants was generated that included a relatively large number of residues that may contribute to binding while the average number and position-specific frequency of amino acid replacements was carefully controlled. As a consequence, screening of a relatively small library revealed (sub−) nanomolar inhibitors. Bioactivity was confirmed in cell culture through a dose-response inhibition assay on the surface of human cancer cells. Taking into consideration the high affinity and selectivity combined with the high general thermodynamic stability of miniproteins, the variants described here may become promising tools for applications in cancer diagnostics. *In vivo* experiments towards tumor targeting with labeled synthetic miniproteins are currently in progress.

## Supporting Information

Figure S1 **Sequences and structure alignment of cystine-knot trypsin inhibitors.** Secondary structure of oMCoTI-II (light brown, pdb: 1ha9, upper left) and SOTI-III (light blue, pdb: 4aor, upper right) is shown as cartoon and cysteine residues are depicted as yellow sticks; protease-binding regions are depicted in red. Cystine-forming residues are marked bold, and the numbering of respective cysteines is according to their appearance in the sequence.
(PNG)

Figure S2 **HPLC trace of MCoTI-variants isolated from seeds of** ***Momordica cochinchinensis*** **.** x marks an unidentified peak. [β-Asp]-MCoTI-II possesses a β-aspartyl residue at position 4. Cyclic miniproteins were isolated from 5 g of homogenized seeds. Extraction was performed using 20 mL aqueous sodium acetate (20 mM, pH 4.5) at ambient temperature for 16 h. The suspension was filtrated and proteins were denatured with 20 mL aqueous acetone (40%, v/v), while the miniproteins remained their native conformation. After removal of acetone under reduced pressure, the suspension was filtrated and the filtrate was purified by semi-preparative HPLC using an axia-packed Phenomenex Luna C18 (250×21.2 mm, 5 µm, 100 Å) column applying linear acetonitrile gradients at a flow rate of 10 mL/min. Isocratic elution (10% eluent B over 5 was followed by a linear gradient of 10→55% B over 30 min.
(TIF)

Figure S3 **Small-molecule inhibitors of matriptase-1 that were used as reference compounds.**
(PNG)

Figure S4 **Matriptase-1 binding analysis of miniprotein variants SOTI Var. 1 and MCoTI Var. 4 via flow cytometry.** (**A**) Sequence of the isolated matriptase-1 inhibitors with randomized residues depicted in the according color. (**B**) Overlay of FACS histograms after labeling of miniprotein-displaying yeast cells with 1 µM of biotinylated matriptase-1 followed by incubation with Streptavidin, R-phycoerythrin conjugate.
(PNG)

Figure S5 **Knottin library design.** Expected distribution of the appearance of amino acid exchanges in loop 1 (red), flanking regions of loop 1 (yellow), and loop 4 (green). The calculation was performed assuming a binominal distribution function.
(PNG)

Figure S6 **Sequence alignments of MCoTI variants isolated from two screening cycles.** Amino acids marked in red are identical to those of the MCoTI-*wt*; amino acids highlighted in red are conserved for all aligned sequences. The blue frames show the consensus of at least two amino acids. The consensus sequence (bottom line) was calculated with a threshold of 0.5. Consensus sequence: upper-case letters indicate sequential identity, lower-case letters illustrate consensus. MCoTI *wt* was taken as lead sequence for the alignment. Sequences that were selected for chemical peptide synthesis and further studies are marked on the right.
(PNG)

Figure S7 **HPLC and MS analysis of folded miniprotein SOTI Var. 1.** (**A**) HPLC trace (10 to 80% B over 20 min) at 220 nm. (**B**) ESI-MS of peptide-containing fraction.
(TIF)

Figure S8 **HPLC and MS analysis of MCoTI Var. 1.** (**A**) HPLC trace (10 to 60% B over 20 min) at 220 nm. (**B**) ESI-MS of peptide-containing fraction.
(TIF)

Figure S9 **CD spectroscopy of the reduced (unfolded) and oxidized (folded) variants of SOTI** ***wt*** **and SOTI Var. 1.** Smoothed with the ‘smooth’ function of Sigma Plot 11.
(TIF)

Figure S10 **CD spectroscopy of the reduced (unfolded) and oxidized (folded) variants of MCoTI** ***wt*** **and MCoTI Var. 4.** Smoothed with the ‘smooth’ function of Sigma Plot 11.
(TIF)

Table S1 **Apparent inhibition constants towards matriptase-1 of the isolated cyclic MCoTI variants.**
(PDF)

Table S2 **Characterization of synthetic miniproteins.**
(PDF)
