# Supplementary figures and images for: Combinatorial Optimization of Cystine-Knot Peptides towards High-Affinity Inhibitors of Human Matriptase-1
Source: PLoS One. 2013 Oct 11;8(10):e76956. doi: 10.1371/journal.pone.0076956 (PMC3795654; doi:10.1371/journal.pone.0076956)

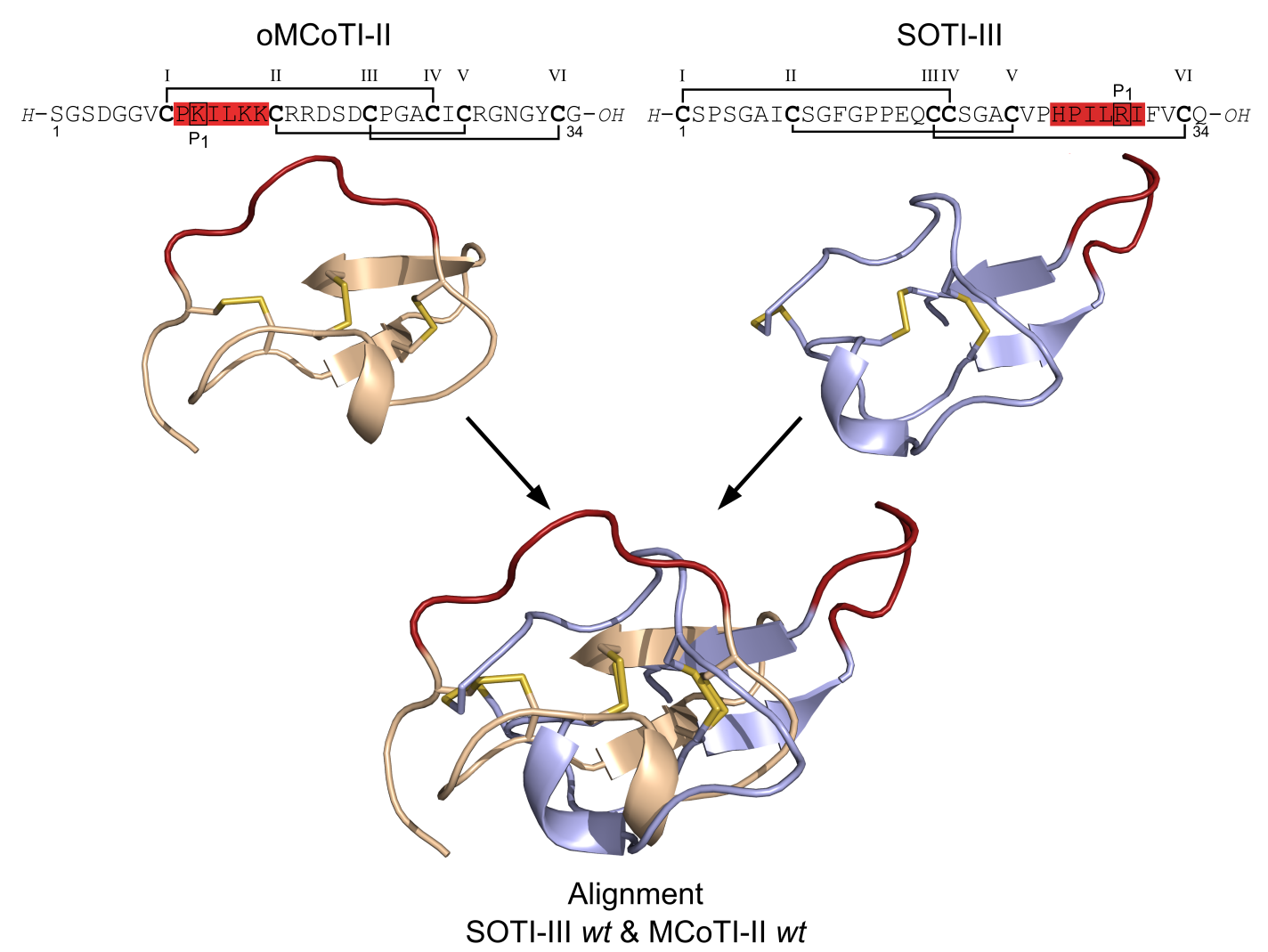

Supplement: Figure S1 — Sequences and structure alignment of cystine-knot trypsin inhibitors. Secondary structure of oMCoTI-II (light brown, pdb: 1ha9, upper left) and SOTI-III (light blue, pdb: 4aor, upper right) is shown as cartoon and cysteine residues are depicted as yellow sticks; protease-binding regions are depicted in red. Cystine-forming residues are marked bold, and the numbering of respective cysteines is according to their appearance in the sequence. (PNG) [file pone.0076956.s001.png]

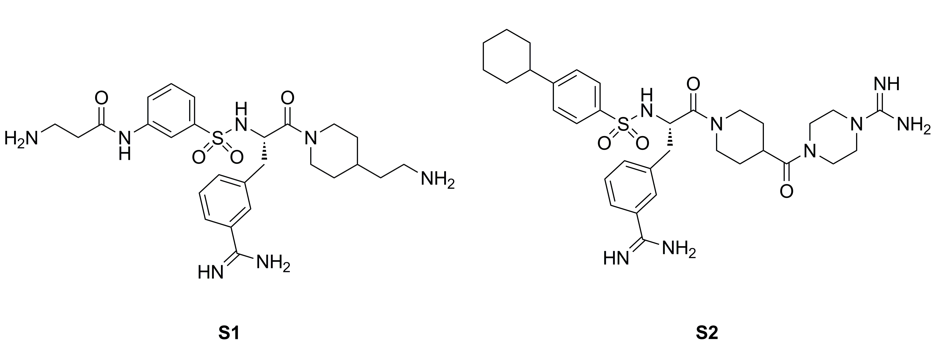

Supplement: Figure S3 — Small-molecule inhibitors of matriptase-1 that were used as reference compounds. (PNG) [file pone.0076956.s003.png]

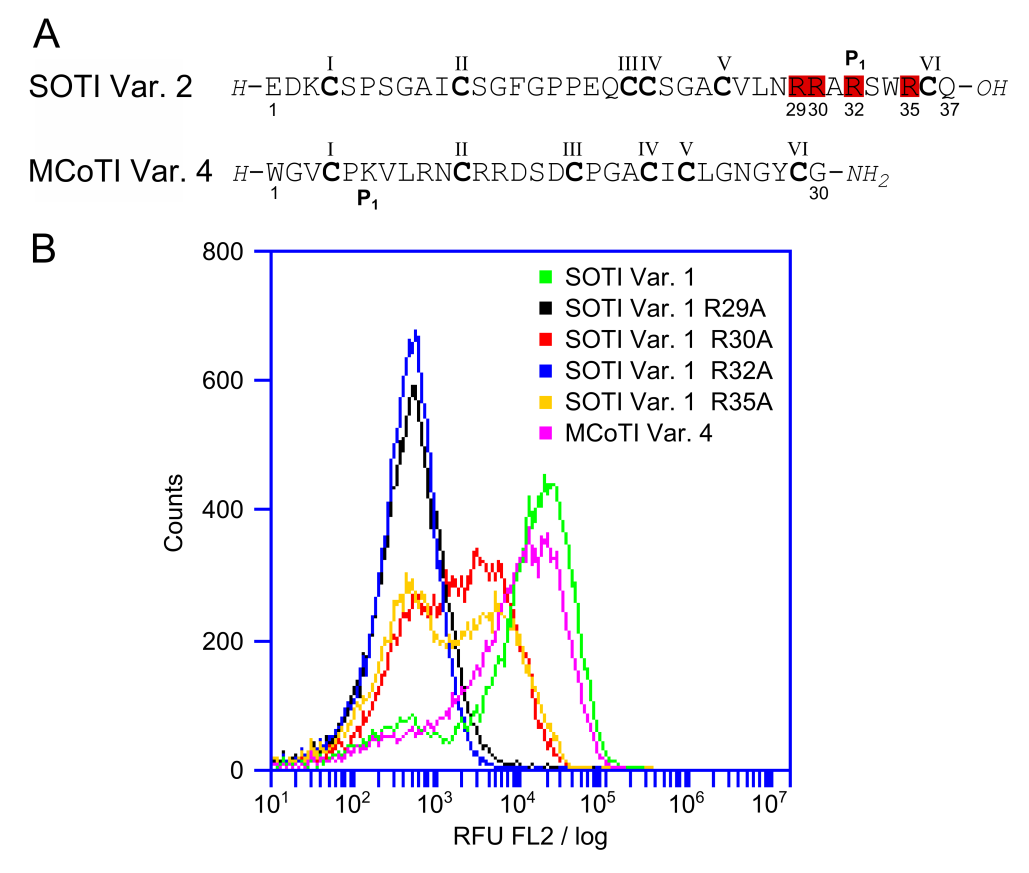

Supplement: Figure S4 — Matriptase-1 binding analysis of miniprotein variants SOTI Var. 1 and MCoTI Var. 4 via flow cytometry. (A) Sequence of the isolated matriptase-1 inhibitors with randomized residues depicted in the according color. (B) Overlay of FACS histograms after labeling of miniprotein-displaying yeast cells with 1 µM of biotinylated matriptase-1 followed by incubation with Streptavidin, R-phycoerythrin conjugate. (PNG) [file pone.0076956.s004.png]

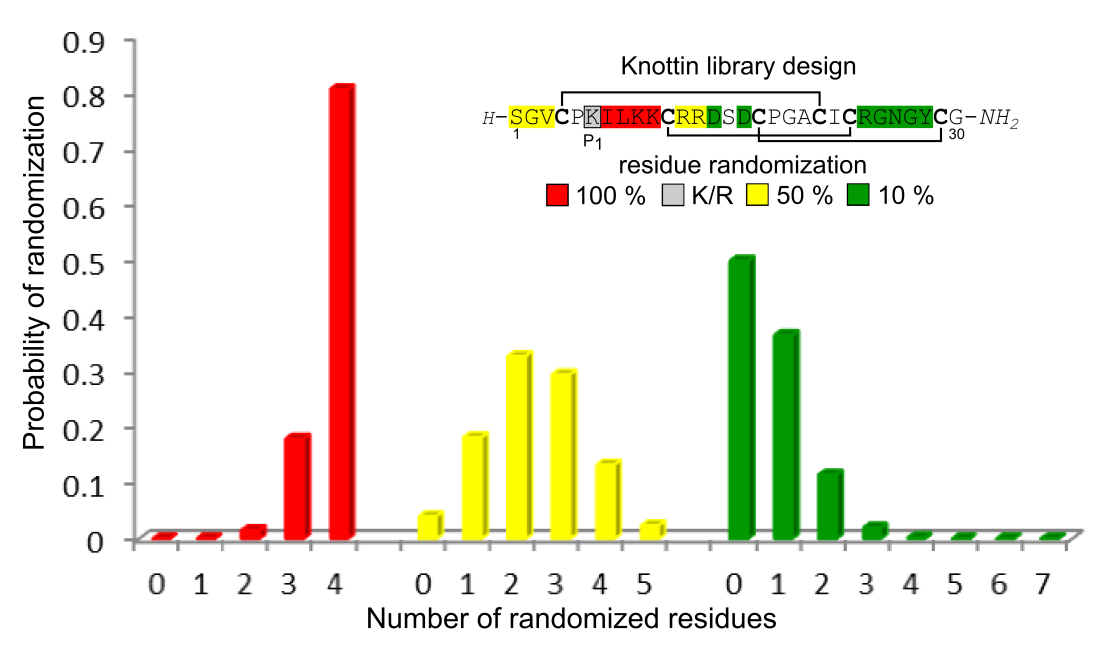

Supplement: Figure S5 — Knottin library design. Expected distribution of the appearance of amino acid exchanges in loop 1 (red), flanking regions of loop 1 (yellow), and loop 4 (green). The calculation was performed assuming a binominal distribution function. (PNG) [file pone.0076956.s005.png]

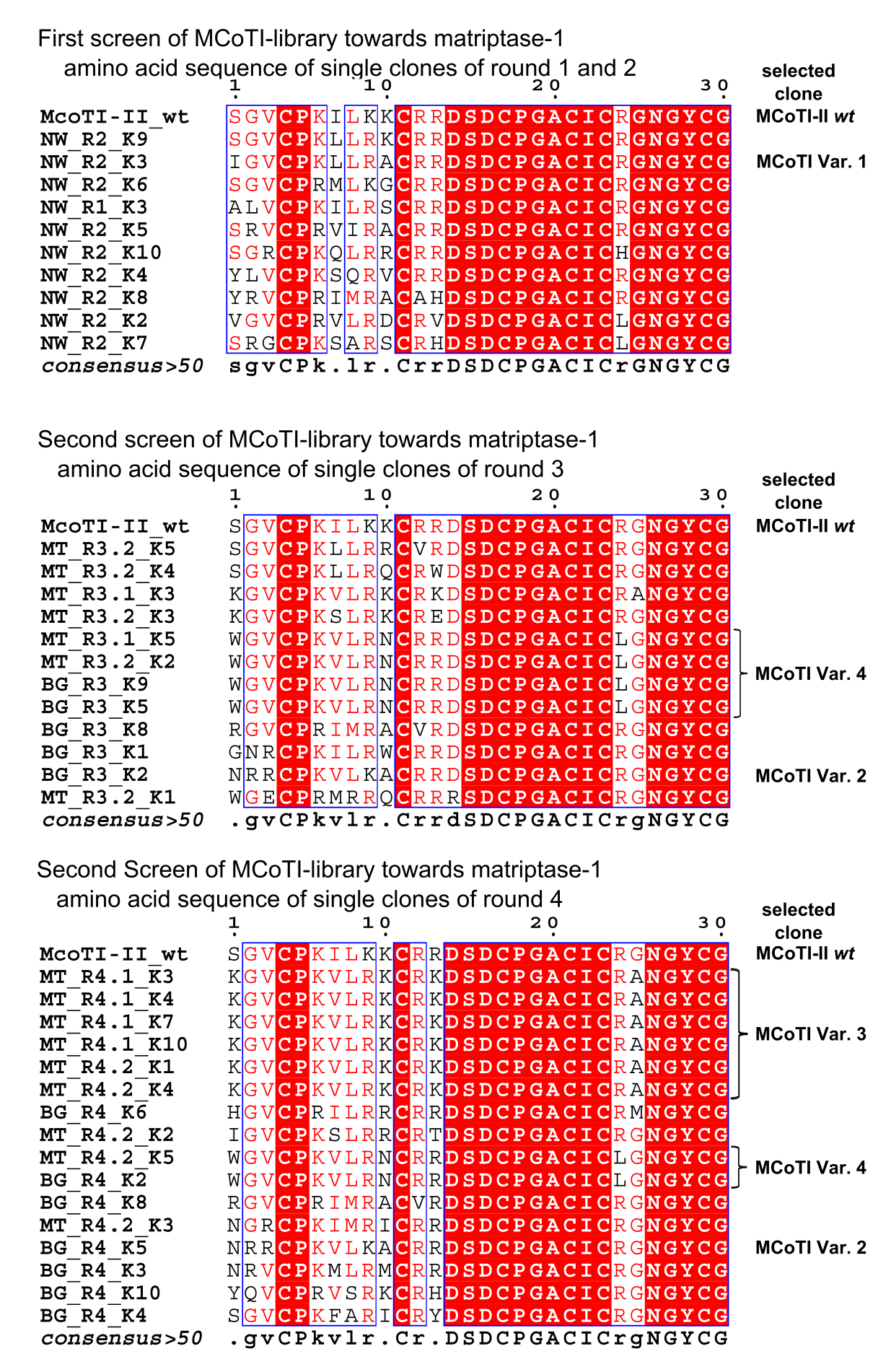

Supplement: Figure S6 — Sequence alignments of MCoTI variants isolated from two screening cycles. Amino acids marked in red are identical to those of the MCoTI-wt; amino acids highlighted in red are conserved for all aligned sequences. The blue frames show the consensus of at least two amino acids. The consensus sequence (bottom line) was calculated with a threshold of 0.5. Consensus sequence: upper-case letters indicate sequential identity, lower-case letters illustrate consensus. MCoTI wt was taken as lead sequence for the alignment. Sequences that were selected for chemical peptide synthesis and further studies are marked on the right. (PNG) [file pone.0076956.s006.png]
