# Supplementary material for: Combinatorial Optimization of Cystine-Knot Peptides towards High-Affinity Inhibitors of Human Matriptase-1
Source: PLoS One. 2013 Oct 11;8(10):e76956. doi: 10.1371/journal.pone.0076956 (PMC3795654; doi:10.1371/journal.pone.0076956)
Supplement: Table S1 — Apparent inhibition constants towards matriptase-1 of the isolated cyclic MCoTI variants. (PDF) [file pone.0076956.s011.pdf]

Table S1: Apparent inhibition constants towards matriptase-1 of the isolated cyclic MCoTI variants.

**Miniprotein**	**Amino acid sequence of cyclic MCoTI variants; inhibitor loop between CysI and CysII is marked bold**	K_i^{app} **(nM)**	**Yield (from 5 g seeds) (mg)**
MCoTI-I	SGSDGGVC**PKILQR**CRRDSDCPGACICRGNGYCG	>1000	1.5
MCoTI-II	SGSDGGVC**PKILKK**CRRDSDCPGACICRGNGYCG	231±12	1.0
oMCoTI-II[a]	SGVC**PKILKK**CRRDSDCPGACICRGNGYCG	276±13	n. d.
MCoTI-II (ß-asp)	SGS(β-D)GGVC**PKILKK**CRRDSDCPGACICRGNGYCG[b]	388±32	1.4
MCoTI-III[a]	(pyro-E)RAC**PRILKK**CRRDSDCPGECICKENGYCG[c]	275±11	1.9

a oMCoTI-II and MCoTI-III possess a linear backbone,b: (β-D) stands for a β-aspartyl bond, c: pyro-E stands for pyroglutamate eliminating cyclization.
