# Supplementary material for: Combinatorial Optimization of Cystine-Knot Peptides towards High-Affinity Inhibitors of Human Matriptase-1
Source: PLoS One. 2013 Oct 11;8(10):e76956. doi: 10.1371/journal.pone.0076956 (PMC3795654; doi:10.1371/journal.pone.0076956)
Supplement: Table S2 — Characterization of synthetic miniproteins. (PDF) [file pone.0076956.s012.pdf]

Table S2: Characterization of synthetic miniproteins.

**Miniprotein**	**HPLC Retention time (min)**	**Calculated masses**	**Measured masses**	**Yield (pure, according to crude product)**
SOTI *wt*	13.92 [a]	M+3H]$^{3+}$: 1278.13<br>M+4H]$^{4+}$: 958.85	M+3H]$^{3+}$: 1278.11<br>M+4H]$^{4+}$: 958.77	10.8 mg<br>23.8 %
SOTI Var. 1	12.78 [a]	M+3H]$^{3+}$: 1319.14<br>M+4H]$^{4+}$: 989.61<br>M+5H]$^{5+}$: 791.88	M+3H]$^{3+}$: 1319.11<br>M+4H]$^{4+}$: 989.54<br>M+5H]$^{5+}$: 791.83	4.5 mg<br>12.5 %
MCoTI *wt*	9.68 [b]	M+3H]$^{3+}$: 1081.26<br>M+4H]$^{4+}$: 811.19<br>M+5H]$^{5+}$: 649.15	M+3H]$^{3+}$: 1081.40<br>M+4H]$^{4+}$: 811.45<br>M+5H]$^{5+}$: 649.30	8.5 mg<br>22.0 %
MCoTI Var. 1	12.40 [b]	M+2H]$^{2+}$: 1576.35<br>M+3H]$^{3+}$: 1051.23<br>M+4H]$^{4+}$: 788.67<br>M+5H]$^{5+}$: 631.14<br>M+6H]$^{6+}$: 526.12	M+2H]$^{2+}$: 1576.20<br>M+3H]$^{3+}$: 1051.08<br>M+4H]$^{4+}$: 788.56<br>M+5H]$^{5+}$: 631.06<br>M+6H]$^{6+}$: 526.09	6.2 mg<br>20.0 %
MCoTI Var. 2	9.49 [b]	M+3H]$^{3+}$: 1089.59*<br>M+4H]$^{4+}$: 817.45<br>M+5H]$^{5+}$: 654.16	M+3H]$^{3+}$: 1089.86<br>M+4H]$^{4+}$: 817.74<br>M+5H]$^{5+}$: 654.45	6.4 mg<br>19.1 %
MCoTI Var. 3	9.39 [b]	M+3H]$^{3+}$: 1065.93<br>M+4H]$^{4+}$: 799.70<br>M+5H]$^{5+}$: 639.96	M+3H]$^{3+}$: 1066.14<br>M+4H]$^{4+}$: 799.93<br>M+5H]$^{5+}$: 640.23	7.2 mg<br>18.2 %
MCoTI Var. 4	13.61 [b]	M+3H]$^{3+}$: 1070.90<br>M+4H]$^{4+}$: 803.43<br>M+5H]$^{5+}$: 642.94	M+3H]$^{3+}$: 1071.00<br>M+4H]$^{4+}$: 803.56<br>M+5H]$^{5+}$: 643.09	5.8 mg<br>14.1 %

a Linear gradient of eluent B from 10 to 80 % over 20 minutes. b Linear gradient of eluent B from 10 to 60 % over 20 minutes.
